# Eco-geomorphic response of the Funafuti Atoll reef flat, central Pacific, to sea-level rise and tropical storms over 120 years (1898–2019)

**DOI:** 10.1098/rsos.252405

**Published:** 2026-10-21

**Authors:** Paul S. Kench, Roger F. McLean, Peter L. Hosking, Murray R. Ford

**Affiliations:** ^1^ Coastal Marine Group, School of Science, The University of Waikato, Hamilton, New Zealand; ^2^ Department of Geography, National University of Singapore, Singapore; ^3^ School of Science, University of New South Wales, Canberra, Australia; ^4^ Faculty of Science, The University of Auckland, Auckland, New Zealand; ^5^ School of Environment, The University of Auckland, Auckland, New Zealand

**Keywords:** reef flats, eco-geomorphology, sea-level rise, tropical cyclones, Tuvalu

## Abstract

This study examines eco-geomorphic changes on the exposed reef flat of Funafuti Atoll over the past 120 years to assess the relative influence of sea-level rise and storm events on reef flat and adjacent environments. Despite a documented sea-level rise of approximately 0.45 m during this period, we find no evidence of long-term structural or ecological alteration on the reef flat itself. We attribute this stability to emergent properties that may buffer against sea-level–driven impacts observed elsewhere. By contrast, significant geomorphic adjustments occurred at the reef flat margins: spur destruction on the seaward edge and increased island elevation landward. These changes reflect cyclone-induced disturbances combined with relative submergence of the reef flat. Notably, this research provides the first empirical evidence linking rising sea levels to increased atoll island elevation. Our findings suggest that future trajectories of reef flats and islands will depend on site-specific interactions among eco-geomorphic inheritance, sea-level rise rates and storm frequency. While these results are broadly applicable to emergent reef flats typical of Indo-Pacific atolls, further comparative studies across diverse reef flat types are essential to refine predictions of atoll resilience under accelerating climate change.

## 1. Introduction

The physical structure of coral reefs underpins most key ecosystem services provided by reefs [1] including dissipation of ocean wave energy and presumed coastal protection [2]; ecological biodiversity [3]; generation of carbonate material for island building and aggregates [4] and fisheries productivity [5]. Reef structure and the services it supports are also widely expected to deteriorate as a consequence of a range of stresses that include climatic change-induced increased frequency of marine heatwaves, ocean acidification, increased intensity of storms, in addition to anthropogenic impacts [6]. However, there have been few studies of the contemporary eco-geomorphic dynamics of reef structure over the past century to provide empirical evidence of such transformations.

While there has been considerable interest in the ecology and morphology of shallow fore reef environments, there has been comparatively less attention on reef flat structure and eco-geomorphic dynamism in response to environmental change [1,7,8]. Coral reef flats are the intertidal or shallow subtidal structural components of a coral reef exposed partially or entirely at low tide, bounded seaward by the reef crest (the transitional zone to the fore reef slope), and leeward either by reef islands, sand aprons or back reef [7]. Varying in width from tens to hundreds of metres, reef flats and their seaward crests are critical structural units that govern wave breaking and dissipation [2,9,10].

Differences in the ecology and surface morphology of the world's 284 300 km^2^ of reef flats [11] are dependent on geographic location, and the interplay between reef growth and sea-level change during their development in the Holocene. Notable differences exist between the reefs of the Caribbean and those of the Indo-Pacific [12–15]. In the Caribbean, near-horizontal reef flats are absent in many locations, and where present are narrow, reflecting continual reef growth response to gradually rising sea level throughout the Holocene [16]. In the Indo-Pacific, near-horizontal intertidal reef flats that are exposed at the lowest tides are widespread. Such reef flats are often described as ‘emergent’; having reached sea-level during the mid-Holocene high stand and becoming exposed (‘emerged’) with the subsequent sea-level fall. In such instances, the living reef communities represent a living veneer over an older (2000–5000 yr) reef framework. Examples include reefs on the Great Barrier Reef [17–19] and the Maldives [20]. By contrast, other reefs such as those at Takapoto atoll in French Polynesia lagged the pace of sea-level change and only caught up to sea-level in the late Holocene as sea level was falling or had stabilized at its contemporary level [21]. The reef flats that form the perimeter of the atoll of Funafuti are also examples of this latter mode of development with platforms that are exposed above low water less than 2000 years old [22–24].

The potential impact of sea-level rise on coral reef flats was one of the first concerns identified in relation to climate change impacts on coral reefs [25]. Specifically, increasing the water depth between the reef flat surface and tide level because of rising sea levels is expected to transform the hydrodynamics of reef flat environments [26–28]. The new ‘accommodation space’ created [29] would potentially stimulate increased at-island wave action and erosion [9,26,28,30], promote landward migration of ecological zones [31], as well as increase the potential for recolonization of reef flat surfaces and new vertical coral and algal growth [7]. However, few examples of these responses to sea-level rise have been documented since sea level started increasing in the post-industrial era.

Examples of new coral growth in response to rapid short-term increases in relative sea level have been described from several locations in the Indo-Pacific, with each related to different causes, including (i) earthquake subsidence in the Solomon Islands [32] and Phuket, Thailand [33], (ii) modifications to reefs from engineering works on Heron Island, Great Barrier Reef [34], and (iii) strong seasonal metocean variations in the Andaman Islands [35]. Moreover, some corals appear to be particularly responsive to sea-level variations, including those of the genus *Porites.* For example, rapid vertical extension of *Porites* microatolls over a 6–8-year period in Palau has been documented that matched the increase in sea level over the same time [36]. Recent colonization of the approximately 5400-year-old senescent reef flat by *Porites* colonies in response to a sea-level rise of 16.2 ± 0.6 cm over 30 years (1987–2016) was also reported at Sanya Bay, northern South China Sea [37].

While these studies provide interesting examples of coral colony response on reef flats to sea-level rise, they do not provide an appropriate analogue for the holistic morphological changes that a reef flat could undergo during more gradual multi-decadal sea-level rise, a time frame across which other changes in boundary conditions such as storm events and marine heat waves would be imbedded. In fact, we are not aware of any field-based study that has documented reef flat changes over the last century. A unique opportunity to undertake such an analysis is provided at Funafuti Atoll, Tuvalu, in the central Pacific (figure 1), owing to the rich temporal dataset available. First, there are detailed ecological and morphological data contained in a precision surveyed plan map of a section of the reef flat undertaken during the third Royal Society Expedition to Funafuti in 1898 [39]. This survey provides a unique baseline that has allowed subsequent changes on the reef flat to be documented. Second, the sea-level record over the past century is well resolved and indicates sea-level rise at Funafuti has been higher than the global average. Global mean sea level (GMSL) has risen at rates of 1.35 mm yr^−1^ from 1901–1990 and up to 3.25 mm yr^−1^ during 1993–2018 [40]. Rates of rise have been higher in the central Pacific, reaching a rate of approximately 5.1 mm yr^−1^ over the 60-year period 1950–2009 centred around Funafuti [38] (figure 1*a*). The sea-level gauge at Funafuti Atoll also indicates a rate of sea-level rise of 4.51 ± 1.2 mm yr^−1^ over the past three decades (based on data from the Permanent Service for Mean Sea Level). These trends correspond to a rate approximately three times that of GMSL and a total increase in sea level of approximately 0.30 ± 0.04 m during that period [38]. It is therefore estimated that sea level has risen by at least 0.45 m between 1901 and the present. Third, although in a low latitude, 8° S, 179° E, Funafuti has been impacted by a number of tropical storms since the late nineteenth century [41] including a major tropical cyclone, Tropical Cyclone (TC) Bebe in October 1972 [42,43] (figure 1*b*). This storm resulted in the formation of a massive coral rubble rampart, approximately 19 km long, approximately 40 m wide and approximately 4 m high along the eastern reef platform of Funafuti, its northern terminus reaching within 50 m of the 1898 surveyed site (figure 1*c*). At the surveyed site, six weeks after the cyclone, storm rubble consisting of ‘white abraded coral plates together with more angular material’ formed discontinuous ‘boulder tracts’ comprising low clast-thick deposits rather than a rubble rampart [43].

**Figure 1.**
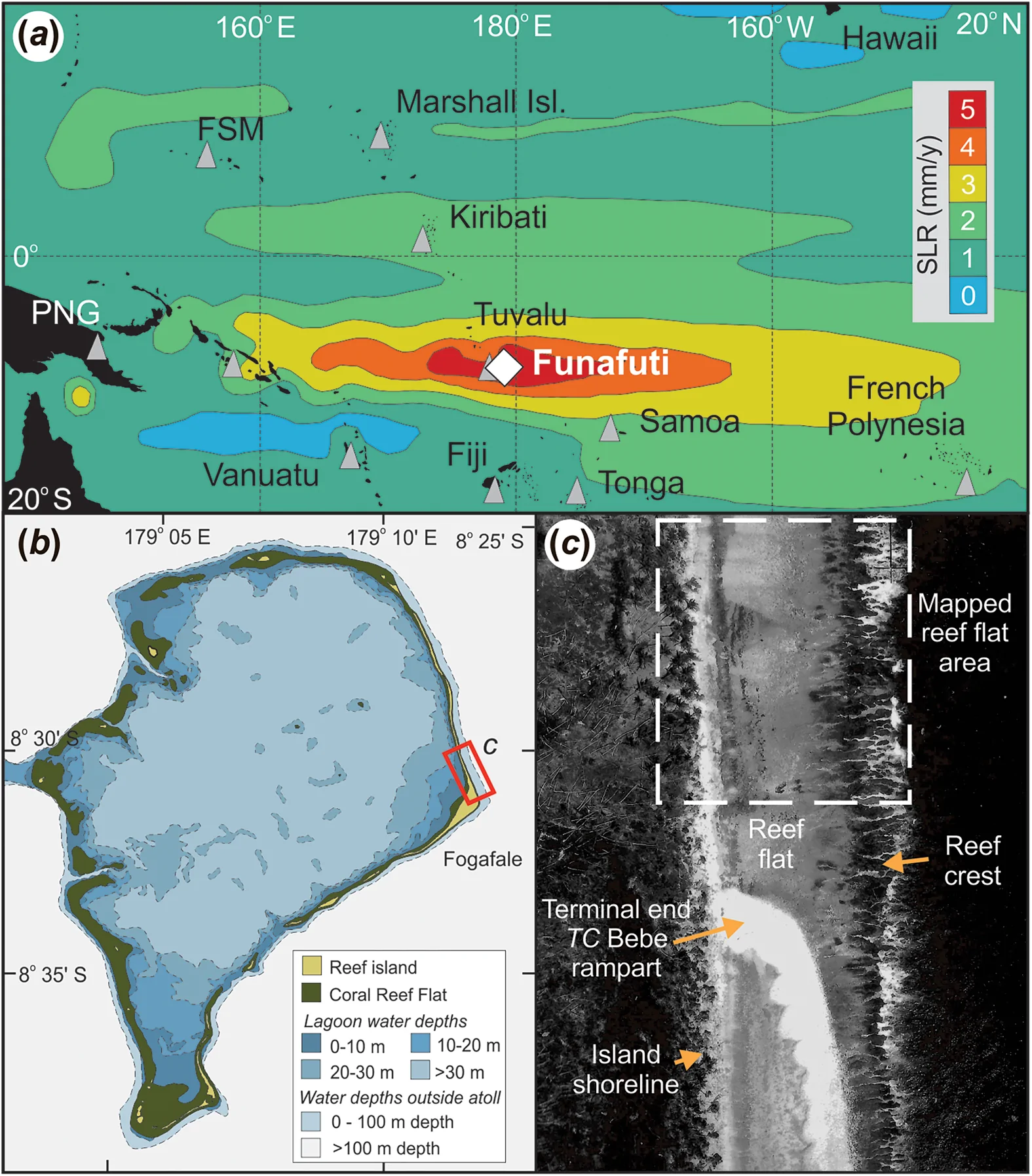
(*a*) Location of Funafuti Atoll and rates of regional sea-level change in the Central Pacific, 1950–2009, after [38]. (*b*) Funafuti Atoll with the reef flat site located north of the eastern elbow of Fongafale Island. (*c*) Vertical aerial photograph of the reef flat site (dashed outline) and surrounds taken six weeks after TC Bebe (October 1972). The site is immediately north of the terminus of the storm rampart that extended continuously along the reef flat on the eastern side to southwestern tip of the atoll (RNZAF Photo 389/58, 18 December 1972).

The combination of these three factors at a single location: (i) a detailed map and description of a section of reef flat in 1898 that serves as an eco-geomorphological baseline, (ii) a local record of sea-level rise over several decades, and (iii) an atoll that has been subject to a number of tropical storms over the last century including a major event in 1972, indicates Funafuti is a unique site to document centennial-scale changes on a contemporary coral reef flat in the context of rapid sea-level rise.

## 2. Material and methods

### 2.1. Field site and study area

Located in the south-west Pacific Ocean, Funafuti Atoll is approximately 25 km north–south and 17.5 km in width and has a perimeter of approximately 71.1 km. The atoll rim has an extensive and near continuous reef flat that is exposed at the lowest tidal level, and that extends 62.7 km (88.2% of rim circumference) enclosing the central lagoon. The atoll rim is punctuated by a number of deep and shallow passages that total 8.39 km in length (11.8% of the periphery) and connect the open ocean and lagoon. The reef flat supports 30 vegetated reef islands, which are accumulations of storm-derived and reef-generated material deposited on the reef flat surface [22,24,44–46]. The presence of islands constrains the width of contemporary reef flats. Along the eastern exposed margin of the atoll, reef flat width between the island shoreline and reef crest ranges from approximately 60 to 100 m. By contrast, where islands are not present, the reef flat width extends up to approximately 300 m.

Like many Indo-Pacific atolls, the reef flat along the eastern and northern coast of Fongafale Island in Funafuti Atoll is characterized by its sub-horizontal morphology, exposure at low tide and ‘barrenness’ of its surface. During the third Royal Society expedition to Funafuti in 1898, a detailed plan of a small area of the reef flat seaward of the expedition's main drilling site and camp was mapped in detail by Finckh [47] (figures 1*c* and 2*a*). The survey area was approximately 150 m long and 100 m wide and extended from the island shore to the reef front (figure 2*a*). Mapping control was provided by several copper plugs that were inserted into the reef flat surface to serve as permanent markers (figure 2*e*). These were later surveyed by another member of the expedition (G. H. Halligan, Hydrographer and Chief Surveyor to the NSW Government) who thought the plugs, which only stuck out by 25 mm, ‘were difficult to find’ and ‘would soon be overwhelmed by growth of the reef’ [48]. Halligan marked the positions more clearly—for ‘future visitors’—inserting a number of cement-filled steel pipes within 20 cm of the plugs (figure 2*d*). The pipes were approximately 0.1 m outside diameter, approximately 0.91–1.22 m in length and sunk to a depth of approximately 0.61–0.76 m into the solid reef, with each pipe standing approximately 0.41 m above the surface. In 1976, one of the authors (R.M.) found all the copper plugs and several of the pipes, the latter now rusted and eroded. These survey marks, which are shown on plate 17 and plate 19 of the expedition's report [39], were used as benchmarks to repeat the plan and profile surveys of the reef flat in 1976, 2004 and 2019.

**Figure 2.**
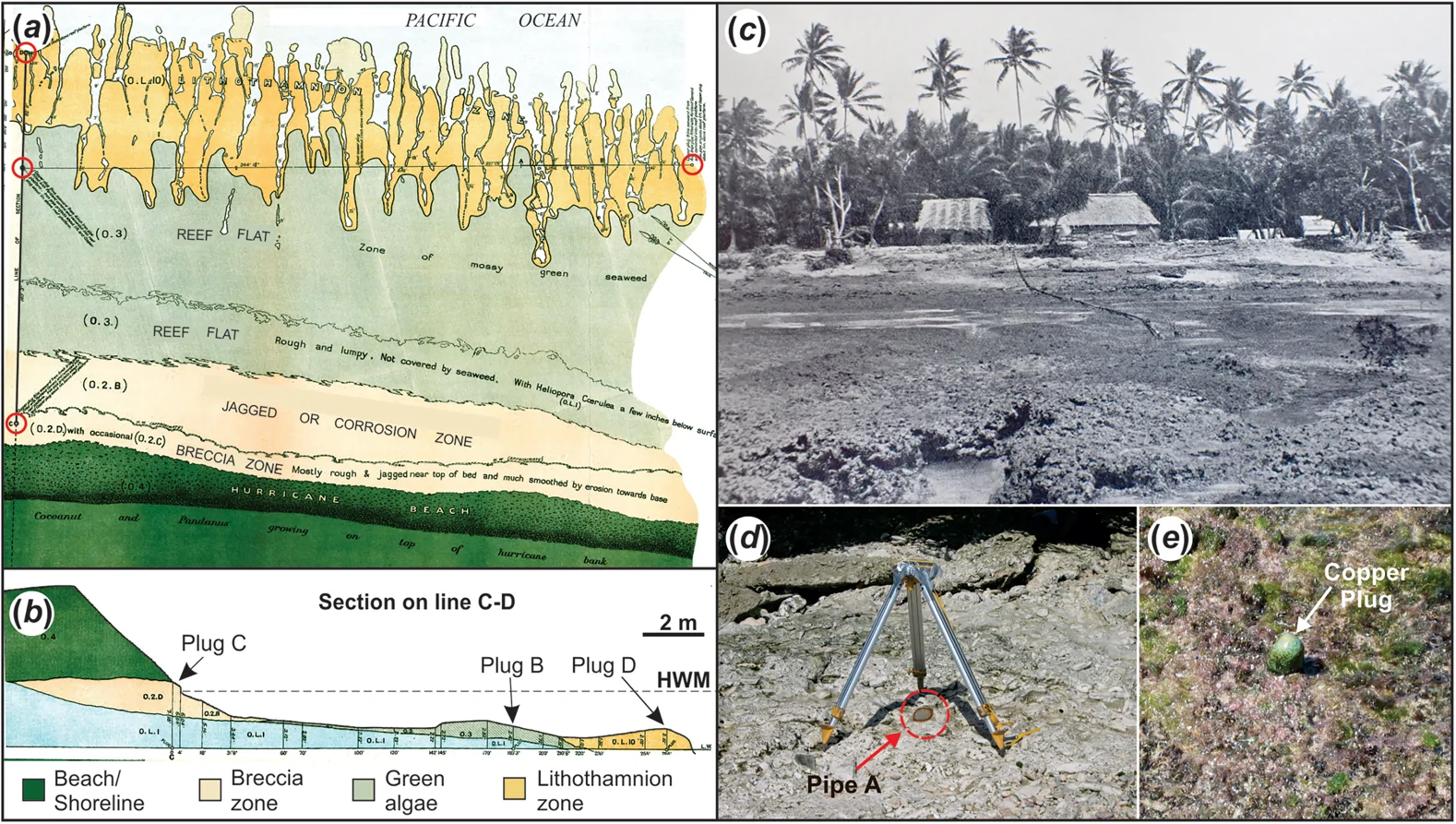
(*a*) Map of reef flat highlighting eco-geomorphic zones from plate 17 [39]. Red circles on the left refer to (from bottom to top) plug C and pipe A, plug B and plug D. (*b*) Cross section from plug C to plug D along the black solid line shown at left of panel (*a*), also from plate 17 [39]. (*c*) View across the central reef flat site to island shore and the 1896–1898 expedition buildings (from Photo Plate E2 p. 79 in [39]). (*d* and *e*) Remnants of original ‘reference markers’ established in 1898 and photographed in 2004; (*d*) remnant of post A and (*e*) the middle copper plug on C–D traverse (*b*).

### 2.2. Initial reef flat description and surveys

The reef flat surface, seaward of the hurricane bank that formed the island shoreline, was divided into four conspicuous eco-geomorphic zones that were mapped on plate 17 [39] and described by Finckh [47, pp. 128–131] (figure 2*a* and summarized in table 1). In general, these earliest observations noted that (i) the reef flat surface was generally smooth and irregular, and lacked large storm blocks, (ii) fleshy green and brown algae was the dominant flora of the central reef flat, (iii) there was little coral growth on the platform surface [47] (figure 2*c*), and (iv) the outer margin comprised a spur and groove (SaG) morphology dominated by living encrusting calcareous algae (*Lithothamnion* sp.).

**Table 1.** Dominant ecological and morphological characteristics of the reef flat based on 1898 surveys (figure 2*a*).

**reef flat zones**	**elevation (m, LWM)**	**substrate characteristics**	**dominant ecological cover**
breccia–corrosion	0.6–1.0	rough cemented rock at landward limit of reef flat comprising angular clasts of coarse sand to coral blocks. Surface is pitted	no living cover
reef flat, seaweed	0.4–0.6	smooth reef pavement surface of fossil calcareous algae	filamentous green and brown algae with foraminifera sand
*Lithothamnion*	0.4–0.7	reef edge spur and groove system. Surface comprises uneven and smooth pavement surface major grooves have alongshore spacing approximately 12 m and length of approximately 20 m	extensive encrusting calcareous algae (*Lithothamnion* sp.). Isolated small corals of *Pocillopra* sp., *Seriatopora* sp. and *Echinometra* occur in cryptic spaces on spurs and in grooves

NB: Names of reef flat zones after plate 17 [39], breccia and corrosion zones combined.

In addition to plan mapping of the ecological characteristics, a topographic profile was surveyed across the reef flat in 1898 (figure 2*a,b*), highlighting eco-geomorphic units across the platform and their elevation with respect to the 1898 low water mark [48] (figure 2*a,b*).

### 2.3. Subsequent monitoring surveys

The topographic profile (plug C to D transect, figure 2*b*) was resurveyed in 1976 using a dumpy level. In 2004, a Trimble differential global positioning system (GPS) survey was undertaken to establish the coordinates of the copper plugs. These coordinates were used to extract the same profile line from the 2019 LiDAR EDM. The LiDAR was captured using a Riegl VQ-1560i sensor. All surveys were subsequently corrected to the 1898 plug C datum 2.18 m above the 1898 low water level. The three profile lines were plotted to examine changes in reef flat topography.

In 2004, a GPS survey of the reef flat area was undertaken that included remapping the eco-morphologic zonation, the inner edge of the spur and groove (SaG) zone and mapping of the location of conspicuous coral boulders and blocks (greater than 40 cm B-axis diameter). The LiDAR survey of the area (2019) was also examined. However, wave conditions at the time of capture were sub-optimal, especially over the northern portion of the SaG zone partially compromising data quality in that area.

### 2.4. Survey uncertainties

Across the 120-year period of analysis, different survey methods have been adopted, and each has different vertical and horizontal uncertainties. Topographic mapping undertaken during the Royal Society expeditions to Funafuti Atoll (1896–1898) was conducted using pace or taped distances and prismatic compass orientation referenced to the position of post ‘A at 7.60 feet above the zero of the tide gauge used on the survey of the atoll, made by Captain Mostyn Field, R.N., of HMS Penguin in 1896’ [48, p. 165]. Horizontal positional uncertainty associated with this approach is estimated to be ±0.6–1.0 m. The topographic cross section in plate 17 (and displayed in figures 2*b* and 3*b*) was undertaken by Halligan using a 12-inch dumpy level and staff, with elevation uncertainty of ±0.2 m. By comparison, the 1976 dumpy level survey has an elevation uncertainty of ±0.1 m, while elevations from the 2019 airborne LiDAR have uncertainties of less than ±0.1 m. The 2004 differential GPS survey of eco-geomorphic zones on the reef flat has a horizontal positional uncertainty of ±1.5 m.

**Figure 3.**
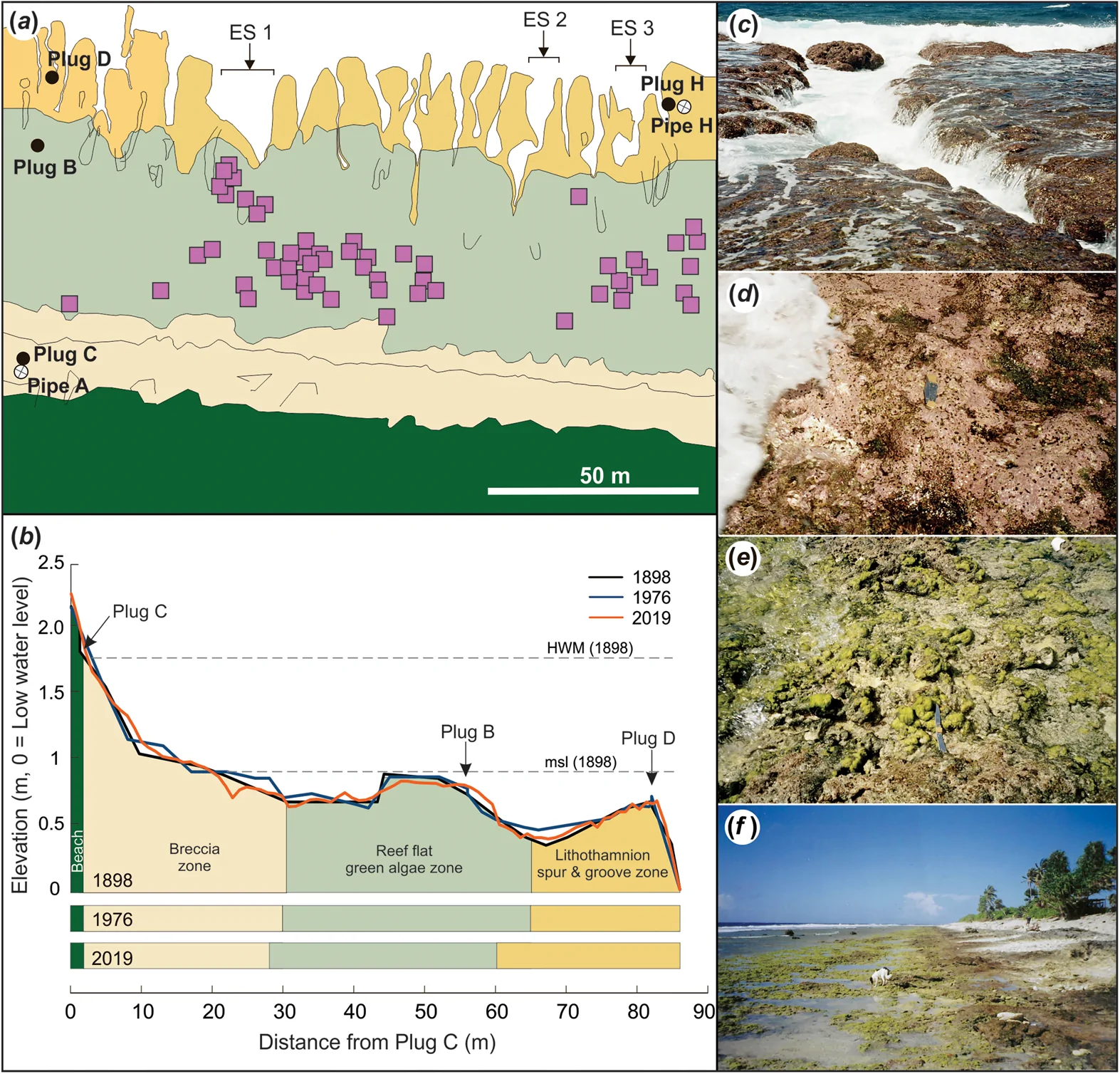
(*a*) Ecomorphological zonation of the reef flat based on GPS survey in May 2004 for comparison with figure 2*a*, black circles denote copper plug locations, purple squares residual reef blocks and boulders initially deposited during TC Bebe, October 1972, ES denotes sites of eroded spurs. Horizontal positioning uncertainty of GPS survey is ±1.5 m. (*b*) Topographic cross section C–D based on three surveys 1898 (figure 2*b*), 1976 (dumpy level) and 2019 (LiDAR EDM) together with ecomorphological zones across the reef flat. Survey elevation uncertainties are ±0.2 m (1898 survey), ±0.1 m (1976 survey) and less than ±0.1 m (2019 survey). (*c*–*f*) Images of cross-shore zones: (*c*) spur and groove; (*d*) *Lithothamnion* zone dominated by pink encrusting coralline algae and filamentous red algae; (*e*) green algae turf zone with foraminifera sand; (*f*) gradient from green algae zone (left), to corrosion and breccia zone, and the mobile white coral rubble of the island's present ‘hurricane’ beach (photos taken in May 2004).

Recognizing these uncertainties, we adopt the following thresholds to determine geomorphic change. Observed spatial discrepancies of less than ±1.5 m, and elevation differences of less than ±0.2 m between survey techniques are considered to fall within the expected uncertainty of survey methods and are not interpreted as evidence of physical eco-geomorphic change.

## 3. Results

### 3.1. Reef flat zonation

Results of the GPS survey of the eco-geomorphic zones in 2004 (figure 3*a*) show that each of the zones identified in 1898 was still clearly present on the reef flat surface. Furthermore, each zone occupied a similar footprint to the 1898 survey (figure 2*a*), with boundaries between the zones also in similar positions over the whole mapped area from the reef front to the island beach. The surveyed topographic profile confirms the comparable pattern for each of the zones across the three surveys, 1898, 1976 and 2019 (figures 2*b* and 3*b*). However, results from 2019 suggest marginal landward movement (approx. 5 m) of the *Lithothamnion* and reef flat green algae zones over the past half-century, which may be indicative of recent recolonization of the reef flat surface.

Comparison of surveys indicates that TC Bebe caused a temporary transition in ecological cover on the central reef flat. While the inner boundary of the *Lithothamnion* zone maintained its position, much of the area located between the *Lithothamnion* and corrosion zones was covered with storm rubble immediately after TC Bebe, which degraded the former green algal surface. However, within a few years, this area was occupied by a filamentous brown-green algal turf that by 2004 was similar to that described by Finckh in 1898 [47]. Within the algal strands, fine-medium sediment was present (figure 3*e*). Analysis of the sediment in 1976 showed it to comprise a mix of abraded foraminifera and *Halimeda* with occasional *Polytrema* and *Echinometra* fragments derived from the SaG and *Lithothamnion* zones rather than being produced *in situ*.

Between the filamentous algae zone and the island beach, the rocky breccia and corrosion zone, described in 1898 as being ‘devoid of life’ [47], was in a similar condition nearly a century later, although in 1976, loose slabby rectangular blocks eroded from the local breccia during the hurricane were strewn over its irregular surface. By 2004, these thin slabs were evident on the island beach. In plan and along the shore, the rocky breccia and corrosion zone had a distinct scalloped and hummocky morphology with mobile coral pebbles and cobbles in the hollows and high points comprised of bare cemented rock (figure 3*f*). Such a pattern was not described by Finckh; nor does the 1898 reef flat map show a rhythmic boundary between the algal and breccia corrosion zones (figure 2*a*).

### 3.2. Spur and groove structure

Comparison of planform maps and surveys indicates minor structural modifications to the spur and groove system as a consequence of *TC Bebe* (figure 3*a*). Six weeks after *the cyclone*, an underwater survey of the SaG area immediately beyond the northern end of the *TC Bebe* rampart (figure 1*c*) was undertaken by Baines *et al.* [43, p. 493], who noted ‘little evidence of damage to the buttresses and spurs’ and that ‘loose rubble fragments’ were found in the shallow grooves whose surfaces were covered with the filamentous algae *Chlorodesmis.* Detailed observations of the same area in September 1976 indicated that three buttresses had been eroded (ES 1, ES 2 and ES 3; figure 3*a*), with two rectangular blocks—the largest with an A-axis of 0.60 m—being wedged into the head of a flask-shaped channel shoreward of the eroded spur (ES 3). Eroded blocks from the other two sites were probably tossed onto the reef flat along with other loose boulders and blocks moved from channels and deeper sources to the intertidal reef flat during TC Bebe. At that time (1976), rubble fragments within the grooves were immobile and covered with encrusting coralline algae similar to that covering channel walls and spur surfaces. *Chlorodesmis* was no longer present and as in 1898, much of the SaG surface was pockmarked with shallow burrows occupied by the echinoid *Echinometra*. Coral growth was limited with small (less than 15 cm) stumpy rosettes of *Pocillopora* and *Stylophora* in crevices and depressions.

### 3.3. Reef flat topography

Comparison of surveyed profile lines across the reef flat indicates that the eco-geomorphic structure has remained remarkably stable over the 120 years of analysis (figure 3*b*). Minor variations in elevation along the profile are within the elevation uncertainty of the survey techniques, and reflect localized differences in the position of the survey points.

### 3.4. Coral block deposits and island shoreline

Recent surveys identified a field of 55 coral blocks (greater than 0.4 m diameter) deposited on the central reef flat surface (figure 3*a*). Analysis of aerial photographs undertaken by the Royal Air Force (RAF) in July 1971 revealed that no reef blocks were on the reef flat 18 months before TC Bebe. However, a repeat set of aerial imagery (RNZAF) flown six weeks after TC Bebe confirm the presence of reef block deposits on the reef flat, which are attributed to the cyclone event. The 55 coral blocks observed during our 2004 site survey are remnants of a more extensive cobble/block unit deposited during TC Bebe. Observations in 1974 also noted the low rubble tract that occupied the middle section of the reef flat in December 1972 (figure 1*c*) became thicker in a shoreward direction and ended in a 0.5 m escarpment [43]. This was not the case in 1976 when the rubble was no longer a coherent ‘tract’ but comprised individual residual blocks and boulders, the smaller cobbles and pebbles being moved landward to form a distinctive beach berm that was subsequently incorporated into the sloping beach profile observed in 2004 (figure 3*f*).

The island shore immediately to the west of the reef flat site has maintained a similar position as in 1898 (figure 3*b*). However, the height of the island ridge top has increased over the past 120 years. Comparison of the elevation of the island ridge in the original 1898 expedition report with the 1976 survey indicates an increase in elevation of approximately 0.69 m. A further increase in island ridge elevation of 0.74 m is identified between the 1976 survey and 2019 LiDAR data. Across the 120-year time frame, these changes total an approximately 1.4 m increase in ridge elevation, well above the elevation uncertainties of the topographic survey methods.

## 4. Discussion and conclusions

### 4.1. Changes to reef flat eco-geomorphology

Over the past century, and into the future, sea-level rise is expected to have severe impacts on the reefs, reef platforms and islands of tropical atolls [6,49,50]. Funafuti Atoll has provided the longest centennial record of reef island changes—using the detailed mapping of all 30 islands on the atoll carried out in 1897 during the second Royal Society expedition to Funafuti Atoll [45]. Changes to islands on other atolls in the Indo-Pacific based on a range of historical data have also been undertaken [46,51–55]. In contrast to atoll islands, there has been no investigation of changes to reef flats over the same time frame, presumably because of the lack of adequate late nineteenth century baselines. However, several studies have suggested that since then, sea-level rise and/or severe storms would modify coral reef flats through increased growth of corals and coralline algae; shoreward migration of ecological zones and changes in biotic communities; damage and/or destruction of exposed reefal structures; erosion and washover of island beaches and ridges and increased inundation and flooding of island surfaces. But there are no centennial-scale observations to test how reef flats have been affected by such potential changes until now.

Our results from Funafuti Atoll show minimal change in eco-geomorphological character of the exposed reef flat that was the subject of a detailed plan and profile survey in 1898 with subsequent comparative surveys in 1976, 2004 and 2019. While this site is small in area (0.15 km^2^), it was noted by Finckh that it is typical of the entire length of the 100–150 m wide platform on the windward northeastern rim of Funafuti Atoll [47, p. 130]. The resurveys documented here show that over 120 years later, the reef flat appears to be in much the same condition in both ecological zonation and topography as described and mapped in 1898. Significantly, this stability in reef structure and ecology has occurred in a region where sea level has risen more than approximately 0.45 m over the last century and where the site has been subject to occasional tropical storms, most notably TC Bebe in 1972. Our detailed comparisons highlight several notable features of eco-geomorphic stability of the reef flat system. First, there has been no substantive across-reef migration of the boundaries of the eco-geomorphological zones (figure 3*a,b*). However, marginal landward movement (less than 5 m) of both the *Lithothamnion* and reef flat green-algae zones between the last two surveys, may signal recent reactivation and recolonization of the reef flat (figure 3*b*). Second, the ecological composition of the *Lithothamnion* and central reef flat green-algae zones is indistinguishable from the earliest accounts, also suggesting ecological stability of these zones. Third, there is no detectable change in reef flat topography. The principal morphological features have retained their elevation and relative position. Subtle differences in elevation between surveys (±0.1 m) reflect localized differences in position of the survey points, and margin of error in survey techniques, rather than material change in reef elevation. The lack of vertical accretion of the reef flat or reef crest is confirmed through the presence of copper plugs emplaced by the 1898 Royal Society Expedition [39]. At that time, it was noted the plugs only stuck out by 25 mm [48]. We found the plugs in similar condition (figure 2*e*) and confirm they had not been overwhelmed by reef growth.

While the fundamental similarity in reef flat conditions between surveys in 1898 and 2019 is a key finding of our analyses, it is also clear there have been several notable changes to the reef flat system, including temporary perturbation to the living cover of the green-algae zone; erosion of at least three spur features at the reef edge; deposition of a field of large (greater than 0.4 m diameter) coral blocks on the central reef flat; and a marked increase in elevation of the island ridge by approximately 1.4 m. We attribute these changes to the role of short-term disturbance events, specifically TC Bebe, which left an indelible geomorphic and ecological imprint on Funafuti Atoll's reefs and islands [42,43].

### 4.2. Tropical Cyclone Bebe impacts ephemeral and persistent

TC Bebe was an extreme event that caused a temporary ecological transition on the reef flat. The event stripped the reef flat of its surficial green-alga cover, exposing a hard rock subsurface comprised of a mixture of calcareous algae and fragmented *Heliopora* and *Porites* corals, similar to those in the corrosion–breccia zone. The immediate post-Bebe surface became covered with the algae *Chlorodesmis* that by 1976 was completely replaced by the present filamentous algal cover. A much longer term structural impact of the cyclone occurred at the reef edge, where there was partial destruction of three spurs in the outer part of the spur and groove system (figure 3*a*). Destruction of these spurs represents immediate loss of reef structure. Material physically removed from these sites was trapped in the groove channels or contributed to storm debris deposited on the reef flat surface [43].

Coral blocks on the central reef flat are the residual clasts of a more extensive cobble/block deposit that formed during TC Bebe. In the years following the cyclone, smaller cobbles and gravels were transported from this deposit under lower energy events and contributed to beach berm development at the adjacent shoreline (figure 3*f*) [56]. Larger blocks were left on the reef flat as residual evidence of the former boulder tract. The last notable geomorphic adjustment in the system has been a marked increase in height of the island berm by approximately 1.4 ± 0.2 m over the 120-year period of analysis. We note that TC Bebe also probably added large clasts directly to the island surface through overwash during the event, as identified in studies at other atoll locations [24,57]. Here, we describe a different mechanism for ridge accretion. TC Bebe generated a large quantity of coarse material, including the reef flat cobble/block deposit, that was subsequently reworked adding sediment volume to the island beach [56]. Successive overwash events have transported these coarse materials to the berm crest, facilitating ridge accretion by a magnitude that is substantially greater (approx. 1.4 m) than the amount of sea-level rise over the past century (approx. 0.45 m). However, the increase in elevation of the island margin has not been accompanied by landward displacement of the shoreline. Both the stability of beach position and the volume of material it contains have been surprise results, though the increase in elevation in response to rising sea level is consistent with recent observations [58], physical laboratory experiments [59] and numerical modelling [60] that indicate overwash mechanisms will enable island margins to increase in elevation.

The persistence of eco-geomorphic characteristics over a 120-year period from 1898 to 2019 suggests there is at least centennial-scale equilibrium in ecological and morphological characteristics on the Funafuti Atoll reef flat. We have shown that impacts of extreme events such as TC Bebe promote short-term perturbations to reef flat ecology followed by a return to its antecedent eco-geomorphic condition. Consequently, we find no evidence that sea-level change over the past century and associated shifts in ocean properties have had a detectable impact on reef flat eco-geomorphology at the field location, and the reef appears to maintain a long-term equilibrium that has filtered extreme events.

The persistence of reef flat conditions despite rising sea levels differs from expected trajectories [6,49]. We attribute this difference to the emergent nature of the reef flat. Independent evidence from Funafuti Atoll indicates reef flats reached sea level approximately 2000–2500 years ago [22,23]. At this time, sea level in the region was up to 1.0 m higher than present [61]. The subsequent fall in sea level in the late Holocene [61] led to their relative emergence, and governed the environmental conditions on the reef flat, which have persisted for two millennia [22,23]. Consequently, we suggest this geomorphic inheritance has imparted a temporal lag on the anticipated onset of reef flat transformations in response to sea-level rise.

### 4.3. Geomorphic changes at reef margin and island

While the reef flat eco-geomorphic system has remained stable across centennial timescales, we have detected geomorphic transformations at both the seaward margin of the reef flat and island shoreline. These geomorphic changes are attributed to the combined role of extreme events and background increase in sea level.

Critical storm impacts were partial destruction of spurs at the reef edge and delivery of coarse materials including large coral blocks to the reef flat surface. Both are medium- to long-term changes in reef topography, which can modify reef hydrodynamics that impact shorelines. First, the loss of spur structures reduces the rate of wave dissipation immediately before the reef crest, therefore increasing the wave energy impacting the reef crest at these sites [62–64]. The inevitable outcome of this change is an increase in wave energy that propagates across the reef flat and exposes the adjacent shoreline to heightened wave impact [28,65]. Counteracting this effect, the presence of storm-deposited coral blocks on the reef flat increases its structural complexity. These additional roughness elements increase frictional dissipation [66] and aid in reducing wave energy across the reef flat. Combined, such adjustments suggest that the impacts of changes in reef structure on reef flat hydrodynamics are not uniformly negative and highlights the importance of resolving the site-specific outcomes of such changes.

In addition to the structural impacts of cyclones that influence wave-reef and island interactions, it must also be noted that we observe no increase in the reef surface despite increasing sea levels. Sea-level data from Funafuti Atoll show there has been an approximately 0.45 m increase over the past century. Given the lack of evidence of vertical reef accretion, this suggests that at Funafuti Atoll, the reef has been subject to relative submergence of an equivalent amount. A number of reports suggest such submergence would have catastrophic consequences for islands [49,50]. Funafuti Atoll represents a contemporary analogue for the scale of such submergence predicted over the coming century. However, not only have the eco-geomorphic zones remained stable, but there has also been no rapid recolonization of the reef surface, nor any sea-level or metocean forced destruction of reef flat structure. Increased water depths across the reef flat can be expected to further increase the wave energy impacting island shorelines [26,67,68]. However, despite assertions that such energy increase will inevitably destabilize islands [69], the shoreline at the Funafuti Atoll site has increased in elevation by approximately 1.4 ± 0.2 m. We conclude that (i) an important role of short-term events is episodic delivery of fresh supplies of coarse material (coral blocks to rubble size) to the reef flat, (ii) as a consequence of sea-level forced increased water depth, and successive storms, heightened wave energy on the reef flat has transported the rubble fraction of this material to the island shoreline, and (iii) increased wave run-up levels and overwash [27], also a consequence of raised sea level, have deposited these coarse sediments at higher elevation along the Funafuti Atoll shoreline. Consequently, our findings indicate that island instability is not an inevitable outcome of sea-level–driven reef flat submergence. A similar conclusion has also been reported in recent studies of island geomorphic response to sea-level change [58–60,70].

A comparison of eco-geomorphic characteristics of the exposed reef flat at Funafuti Atoll over the past 120 years provides new insights on the relative influence of rising sea levels and storms on the geomorphic trajectory of reef flats and adjacent environments. Significantly, we find no evidence of long-term structural or ecological change on the reef flat, despite sea-level rise of approximately 0.45 m over the past century. We attribute this stability to its emergent properties, which may delay the onset of sea-level-driven impacts reported elsewhere. In contrast to the stable reef flat, geomorphic changes were detected at the seaward (destruction of spurs) and landward (increased island elevation) margins of the reef flat. These changes resulted from cyclone-driven impacts and the relative submergence of the reef flat owing to rising sea level. We present the first empirical evidence of the effect of sea level in increasing atoll island elevation. It is apparent that future trajectories of atoll reef flats and islands will depend on the site-specific combination of eco-geomorphic inheritance, rate of sea-level change and storm occurrence. We note that our findings are applicable to emergent reef flats that are typical of Indo-Pacific atolls, but stress that further comparative analyses are necessary from other reef flat types and sites.

## Data Availability

Topographic survey data and global positioning system (GPS) mapping data of the Funafuti reef flat are available in the Dryad repository [71].
